# Immune Cell Infiltration Analysis Demonstrates Excessive Mast Cell Activation in Psoriasis

**DOI:** 10.3389/fimmu.2021.773280

**Published:** 2021-11-23

**Authors:** Yongjun Zhang, Yanqiang Shi, Jingxia Lin, Xuefei Li, Bin Yang, Jiajian Zhou

**Affiliations:** Dermatology Hospital, Southern Medical University, Guangzhou, China

**Keywords:** psoriasis, inflammatory, immune cell niche, mast cell, activation.

## Abstract

Psoriasis represents multiple inflammatory processes and exaggerated physiological responses to epithelial damage by innate and adaptive immune components, thus it is critical to compare the immune cell niche in disease and healthy skin. Here, we inferred the proportions of different immune cell types in psoriatic and healthy skin using the CIBERSORT algorithm with expression profiles as input. As a result, we observed a dramatic change of immune cell profiles in psoriatic skin compared with healthy skin. Interestingly, the resting mast cells is almost eliminated in psoriatic skin. In contrast, the activated mast cells are enriched in psoriatic skin, indicating that mast cells activation may play an important role in psoriasis pathogenesis. In addition, we found that the proportion of the resting mast cells gradually come back to the normal level in lesioned skin upon etanercept treatment, suggesting that mast cells play a critical role in immune cell niche maintenance. Further experiments validated a significant decrease in mast cell population and an excessive mast cell activation in psoriatic skin compared with healthy skin. In conclusion, our integrative analyses of the immune cell profiles and the corresponding marker genes expression provide a better understanding of the inflammation response in psoriasis and important clues for clinical applications.

## Introduction

Psoriasis is a chronic inflammatory skin disorder, which affects more than 10% of people worldwide (1–3). It dramatically reduces patients’ life quality and usually is recurrent under certain stimulants, such as skin trauma, infection, stress, and certain medications, including interferon, beta blocker, and lithium (4). Psoriasis represents multiple inflammatory processes and exaggerated physiological responses to epithelial damage by innate and adaptive immune components (4, 5). Recent studies showed a dramatic change of immune cell atlas between inflamed and healthy skin by leveraging the innovative single-cell RNA-seq (scRNA-seq) technologies (6–8). For instance, Cheng et al. demonstrated the enrichment of a CD1C^+^CD301A^+^ myeloid dendritic cell population in psoriatic epidermis using scRNA-seq (6); Reynolds et al. observed the cellular reprogramming of macrophage in psoriatic lesional skin (7). Furthermore, Liu and his colleagues identified 11 transcriptionally diverse CD8^+^ T-cell subsets in psoriatic and healthy skin using SMART-seq technology. They found two non-exhausted IL-17-producing CD8^+^ (Tc17) cell subsets with diverse metabolic characteristics (8). These studies illustrated the dynamism of cutaneous immunity in psoriasis and provided opportunities for targeting pathological developmental programs in inflammatory skin diseases (7, 8). However, the sample size is relatively small in those studies and the single-cell suspension preparation protocols limit a high resolution of immune cell map in inflammatory tissues, which is crucial for dissecting molecular mechanisms triggering the pathogenesis of psoriasis (9). Therefore, further investigations on immune cell infiltration in psoriatic plaque using computational and experimental methods are required to understand the mechanisms of psoriasis pathogenesis and improve therapeutic efficacy.

Previous studies demonstrated that scRNA-seq is not well suited to characterizing cell-type proportions in solid tissue because the cell suspension preparation pipeline is biased towards certain cell types (10). For instance, it is a significant challenge to perform single-cell analysis of neutrophils and other granulocytes. Because neutrophils (and other granulocytes) have low RNA content, high sensitivity to degradation and high levels of RNases, it leads to extremely low capture efficacy in current single-cell RNA-seq protocols (11, 12). The cutting-edge computational tools have been developed to deconvolve immune cell type proportions by bulk cell transcriptome data using cell type-specific gene expression profiles, such as CIBERSORT, BSEQ-sc, EPIC and TIMER (13–16). Although these methods infer immune cell proportions based on the expression profiles of a set of cell type-specific marker genes, they provide a convenient way to study the dynamics of different cell types in a large sample cohort under a specific disease condition (17). In addition, previous reports have generated a large amount of valuable gene expression profiles in psoriatic lesional skin, healthy skin and psoriatic non-lesional skin upon clinical therapies (18, 19). Therefore, a systematic analysis of immune cell proportion in psoriatic lesional and non-lesional skin will help dissect the functions of different immune cell types and the immune cell homeostasis in psoriasis.

In the present study, we first confirmed the dramatic changes of the transcriptome in psoriatic lesional skin; then we inferred the proportions of 22 immune cell types in psoriatic lesional, non-lesional and healthy skin using CIBERSORT with expression profiles as input. As a result, we observed a dramatic change of immune cell profiles in psoriatic skin compared with healthy skin. Interestingly, the resting mast cells are almost eliminated in psoriatic skin. In contrast, we observed that the activated mast cells are enriched in psoriatic skin, indicating the mast cells activation may play an important role in psoriasis pathogenesis. Intriguingly, we found that the proportion of the resting mast cells gradually returned to the normal level in psoriasis lesional skin upon etanercept treatment. Further experimental validation showed a significant decrease in the mast cell population and a large proportion of the mast cells are activated in psoriatic skin. In summary, our integrative analyses of the inferred immune cell proportions and immune cell marker genes expressions provide a better understanding of the immune response in psoriasis and important clues for clinical application.

## Methods and Materials

### Expression Data Analysis

A set of CEL files was downloaded from GSE13355, including 58 psoriatic patients and 64 normal healthy controls (180 samples in total) (18). Then, we obtained the differential expressed genes (DEGs) in psoriasis lesioned compared with healthy controls using this dataset with standard microarray analysis protocol (15). To analyze the expression dynamics and cell proportion changes upon etanercept treatment, the raw CEL files were obtained from GSE41664, including psoriatic lesional skin upon etanercept treatment for 0 day, 1 week, 2 weeks, 4 weeks and 12 weeks (19). Then, CEL files were analyzed using the standard microarray analysis procedure (15). Firstly, raw CEL files were annotated by hgu133plus2hsentrezgcdf and org.Hs.eg, then the gene expression level was normalized by MAS5 algorithm (20). Finally, the differential expressed genes (DEGs) were determined in psoriatic compared with non-lesional or healthy skin using p-value < 0.05 and log2 Fold Change > 1 as a cutoff. Regarding RNA-seq data derived mast cells with/without IgE-FcϵRI cross-linking treatment, the preprocessed expression file with FPKM value was downloaded from GSE125887 (21), then DEGs were determined using log2 Fold Change > 1 as a cutoff. The detailed information of those datasets has been included in **Supplementary Tables S1**, **S2**.

### Gene Set Enrichment Analysis

The DEGs were submitted to DAVID (https://david.ncifcrf.gov/) gene functional annotation system for Gene Ontology (GO) and Kyoto Encyclopedia of Genes and Genomes (KEGG) enrichment Analysis (22). To further confirm the enrichment results, we applied Gene Set Enrichment analysis (GSEA) (23) to the whole transcriptome using the ranked fold change of expression level between psoriasis and healthy subjects with adjusted p-value < 0.05 and Enrichment Score (ES) > 2 as a cutoff.

### Estimation of Immune Cell Proportions Using Expression Profiles

The immune cell proportions were inferred using the CIBERSORT R package (https://cibersort.stanford.edu/download.php) with inputs of the normalized expression matrix of psoriatic and healthy skin and the expression of a reference signature genes (LM22, https://cibersort.stanford.edu/download.php) (15, 24). Then, a vector represented the proportion of 22 immune cell types was obtained, which will be used in the downstream heatmap and clustering analysis. Briefly, CIBERSORT employs a novel approach named linear support vector regression (SVR) to deconvolve the mixture expression matrix into cell types proportions and the machine learning algorithm is highly robust with respect to noise (25). Next, SVR performs an adaptive feature selection based on the predefined expression matrix of a signature gene set to deconvolve a given mixture, then the final deconvolution is determined by considering the expression of multiple signature genes (15).

### Principle Component Analysis (PCA) and Heatmap Analysis

The gene expression profiles and immune cell proportions of all samples were visualized using ClustVis (https://biit.cs.ut.ee/clustvis/) (26). The PCA was also performed on the datasets of psoriasis and healthy skin using ClustVis, and the dataset of the healthy skin and psoriatic lesioned skin upon etanercept treatment.

### Human Skin Biopsies and Immunofluorescence Assay

Skin biopsies were obtained from psoriasis patients and healthy donors under ethical approval in the Department of Dermatology, Dermatology Hospital of Southern Medical University. All participates have signed the informed consent. Skin sections were deparaffinized by dewaxing solution and rehydrated by gradient alcohol. Antigen retrieval was conducted in microwave oven with citrate buffer (pH = 6). The slides were blocked with goat serum for 30 min at 37°C, then the slides were incubated with rabbit anti-c-Kit antibody (1:300 dilution; Abcam, ab2378) and mouse anti-mast cell tryptase antibody (1:300 dilution; Abcam, ab178527) at 4°C overnight. Next, the slides were incubated with the matched fluorescent secondary antibodies (Abcam) for 30 min at 37°C. Finally, the fluorescence images were captured with a confocal microscope (Nikon A1+, Japan). Because no experimental validated specific marker gene may be used to distinguish activated and resting mast cells, the observed cell was defined as activated mast cells, if it is with positive c-Kit and Tryptase double-positive staining and a spindle-shaped morphology. We then calculated the percentage of activated mast cells according to five independent fields in normal and psoriatic skin, respectively.

### Toluidine Blue Staining

Mast cells with metachromatic granules were quantified by toluidine blue staining according to the manufacture’s instruction (#BA4125, BASO) in tissue sections derived from psoriasis and healthy donors. The quantification of mast cells was evaluated in 5 random non-overlapping fields of view and the total number of mast cells per mm^2^ was averaged for each sample.

## Result

### Inflammatory Pathway Activation Indicates the Tremendous Changes of the Immune Cell Niche in Psoriatic Skin

**Figure 1:**
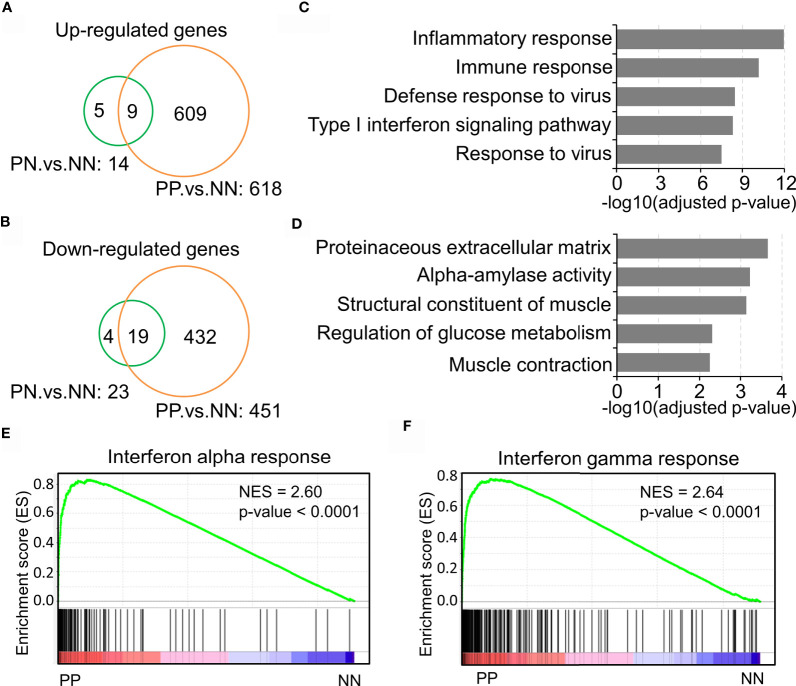
Inflammatory pathway activation indicates the tremendous changes of the immune cell niche in psoriatic skin. **(A, B)** Up-regulated genes **(A)** and down-regulated genes **(B)** in lesion skin of psoriasis (PP) compared with non-lesion skin of psoriasis (PN) and healthy subjects (NN). **(C, D)** Gene ontology (GO) enrichment analysis of up-regulated genes **(C)** and down-regulated genes **(D)**. **(E, F)** Gene Set Enrichment Analysis (GSEA) demonstrates the enrichment of interferon-alpha response **(E)** and interferon-gamma responses **(F)** in psoriatic skin. PP represents lesion skin of psoriasis; PN represents non-lesion skin of psoriasis; NN represents the skin of healthy subjects.

Previous studies have demonstrated that the inflammatory pathways are activated in psoriatic skin compared with healthy skin (19, 27, 28). The present study intended to confirm the transcriptional changes associated with immune response in psoriatic skin by comparative analysis of a large-scale transcriptome dataset with 180 skin biopsy samples. As a result, we obtained 609 up-regulated genes and 432 down-regulated genes in lesional skin of psoriasis (PP) compared with non-lesional skin of psoriasis (PN) and health subjects (NN) (**Figures 1A, B** and **Supplementary Table S2**). Particularly, only a few DEGs in PN *versus* NN are shared by PP *versus* NN, indicating a dramatic change of biological processes in the PP group (**Figures 1A, B**). Gene ontology analysis demonstrated that the up-regulated genes are significantly enriched in the inflammatory response, immune response, defense response to virus and type I interferon signaling pathway, while the down-regulated genes are enriched in proteinaceous extracellular matrix and metabolism pathways (**Figures 1C, D** and **Supplementary Table S2**), suggesting a distinct immune cell niche in psoriatic compared with healthy skin. Further Gene Set Enrichment Analysis (GSEA) showed that interferon-alpha response and interferon-gamma response are significantly elevated in the PP group, confirming the dramatic immune responses in psoriatic skin (**Figures 1E, F**). In summary, our analyses depicted the elevated inflammatory pathways in psoriasis, indicating the tremendous changes of immune cell niche in psoriatic skin.

### Distinct Immune Cell Infiltration in Psoriatic and Healthy Skin

**Figure 2:**
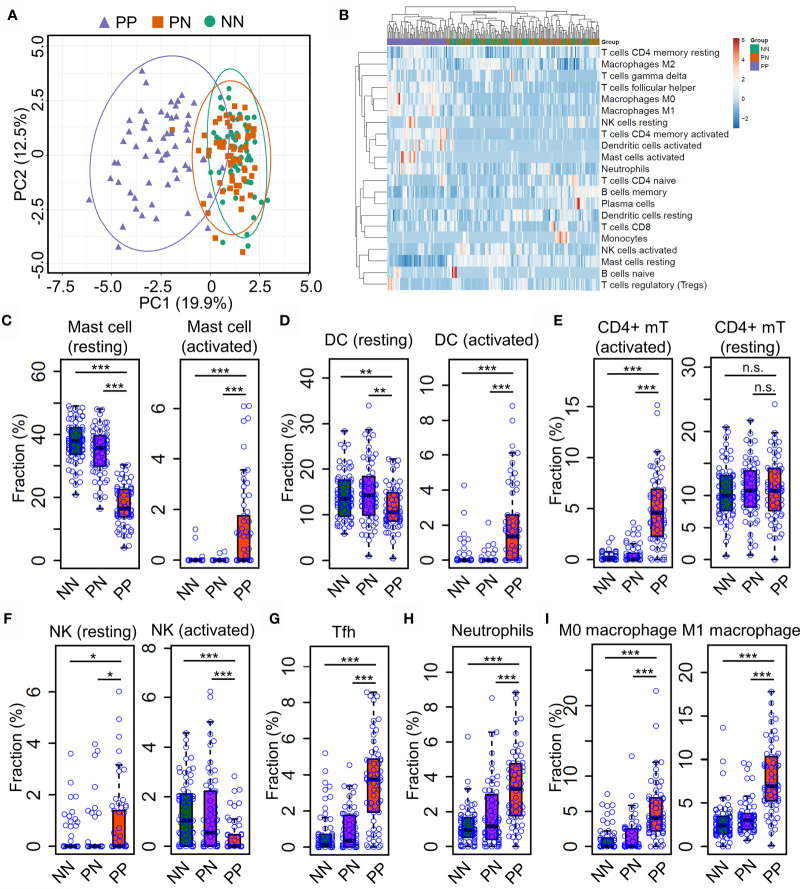
Distinct immune infiltration landscape of psoriatic and healthy skin. **(A)** Principal Component Analysis (PCA) of the fraction profiles of immune cells in lesion skin of psoriasis (PP) compared with non-lesion skin of psoriasis (PN) and health subjects (NN). Purple represents lesion skin of psoriasis; orange and green represent non-lesion skin of psoriasis (PN) and health subjects (NN), respectively. **(B)** hierarchical clustering analysis of the fraction profiles of immune cells in PP compared with PN and NN. **(C–F)**. compare the fraction of resting or activated mast cells **(C)**, dendritic cells **(D)**, CD4+ memory T cell **(E)** and natural killer cells **(F)** in PP with PN and NN. **(G–I)**. Compare the fraction of follicular helper T cells **(G)**, neutrophils **(H)**, macrophage M0 and M1 **(I)** in PP with PN and NN. *P < 0.05; **P < 0.01; ***P < 0.001; n.s, not significant.

Gary R. et al. recently showed that Th17 cells (expressing IL17A, IL17F, IFNG, IL22, and IL26) and type 2 macrophage (M2) are significantly enriched in lesional skin of psoriasis (7); Gaofeng W. et al. showed that Psoriatic Microenvironment (PME) score could predict the treatment response of psoriasis patients (29), these studies provide insights into the pathogenic research of psoriasis. However, a systematical investigation of immune cell niche alternations in psoriatic skin requires further investigations. To this end, we leveraged the CIBERSORT algorithm to predict immune cell infiltration in PP, PN and NN groups using gene expression profiles. Briefly, CIBERSORT employs a novel approach named linear support vector regression (SVR) to deconvolve the mixture expression matrix into cell types proportions by considering the expression pattern of multiple signature genes (15). As a result, PCA analysis demonstrated that the immune cell infiltration of PP is distinct from PN and NN groups (**Figure 2A**), suggesting that the immune cell alternations may lead to chronic inflammation in psoriatic skin. We then sought to investigate the infiltration dynamics of different immune cell types using hierarchical clustering analysis. Interestingly, activated mast cells, activated dendritic cells, activated CD4+ memory T cells, macrophage M0, macrophage M1 and neutrophils are enriched in PP compared with PN and NN groups (**Figure 2B** and **Supplementary Table S3**). Particularly, we found that the proportion of the resting mast cells is decreased in PP, while the activated mast cell is increased compared with PN and NN. Meanwhile, we observed a similar change in dendritic cells, indicating that mast cells, dendritic cells and CD4+ memory T cells are activated for defense the stimulus from the internal and external environment (**Figures 2C–E**). Intriguingly, the proportion of the activated nature killer cells is decreased in PP compared with PN and NN, while the proportion of resting nature killer cells is increased (**Figure 2F**). In addition, the proportion of T helper cells, neutrophils, macrophage M0, macrophage M1 are significantly enriched in PP compared with PN and NN groups (**Figures 2G–I**). Our analyses demonstrated a distinct immune infiltration landscape of psoriatic lesional skin compared with non-lesional and healthy skin, highlighting mast cell activation may play a crucial role in psoriasis pathogenesis.

### Differential Expression of Immune Cell Marker Genes Confers Niche Alternations in Psoriatic and Healthy Skin

**Figure 3:**
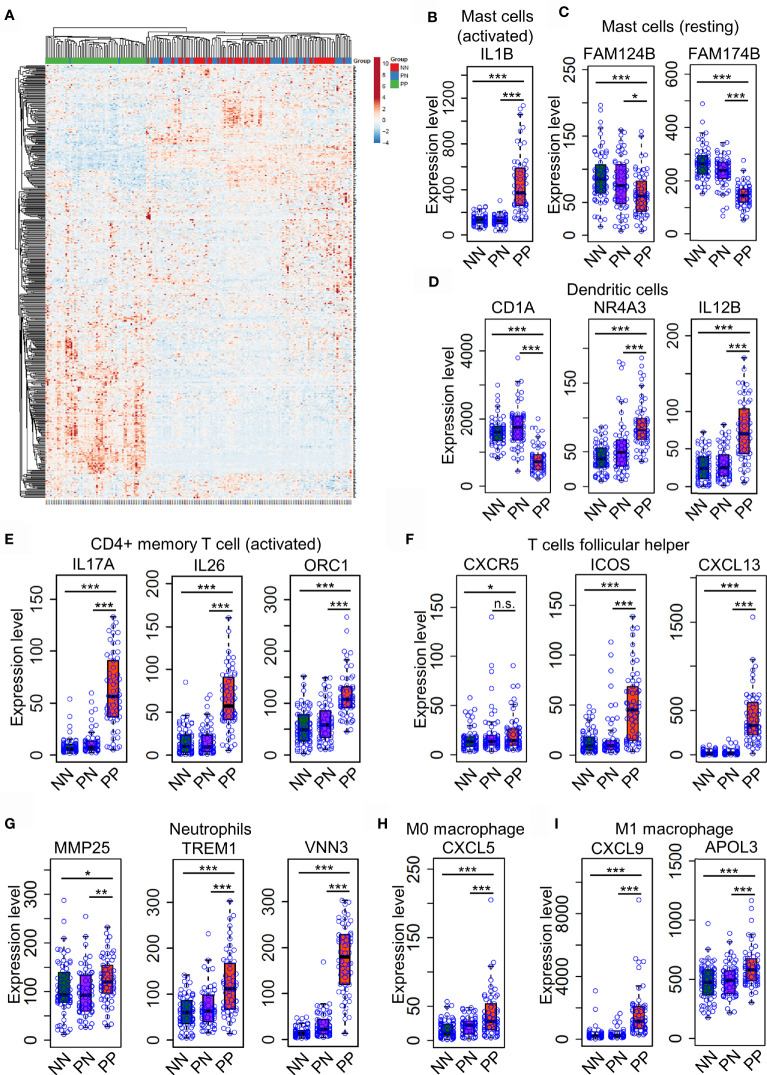
Differential expression of immune cell marker genes confers niche alternations in psoriatic and healthy skin. **(A)**. a hierarchical clustering analysis of marker genes associated with immune cells in the PP group compared with PN and NN group. **(B–H)**. the marker genes expression in mast cells **(B, C)**, dendritic cells **(D)**, activated CD4+ memory T cells **(E)**, follicular T helper cells **(F)**, neutrophils **(G)**, macrophages M0 **(H)** and M1 **(I)** are with higher expression level in PP compared with PN and NN. *P < 0.05; **P < 0.01; ***P < 0.001; n.s., not significant.

Distinct expression profiles of different cell type markers determine the infiltration of the immune cells of a particular environment (29, 30). Thus, we intended to investigate the dynamic expression of 547 marker genes specific in different immune cell types (**Supplementary Table S4**). As a result, the hierarchical clustering analysis demonstrated that the expression pattern of marker genes associated with immune cells is distinct in the PP group compared with PN and NN (**Figure 3A** and **Supplementary Table S4**). A large proportion (202, 38.5%) of marker genes associated with activated immune cells are dramatically up-regulated in PP, conferring an immune cell niche alternation in psoriasis (**Figure 3A**). We then selected signature genes for leukocyte subsets by analysis of LM22 dataset with the similar method described in the CIBERSORT study (15); a signature gene is defined if it is differentially expressed compared with the remaining cell types, resulting in 268 genes for 22 leukocyte cell types (**Supplementary Table S6**). Particularly, the mast cells makers IL1B is significantly up-regulated in PP, while FAM124B and FAM174B is down-regulated in PP, indicating the mast cells are indeed activated in psoriatic skin and they may play a role in the progression of psoriasis (**Figures 3B, C** and **Supplementary Figure S2A–C**). We further observed that the activated marker genes of dendritic cells (CD1A, NR4A3and IL12B) and activated CD4+ memory T cells (IL17A, IL26 and ORC1) are activated in PP compared with PN and NN (**Figures 3D, E**). In addition, the marker genes for follicular T helper cells (CXCR5, ICOS and CXCL13), neutrophils (MMP25, TREM1 and VNN3), macrophage M0 (CXCL5) and macrophage M1 (CXCL9 and APOL3) are significantly up-regulated in PP compared with PN and NN (**Figures 3F–I** and **Supplementary Figures S1**, **S3A–F**). In summary, the analyses of gene expression profiles again confirm the dramatic alternations of immune cell infiltration landscape in psoriatic skin compared with healthy skin.

### Mast Cell Homeostasis Disruption May Contribute to Skin Inflammation

**Figure 4:**
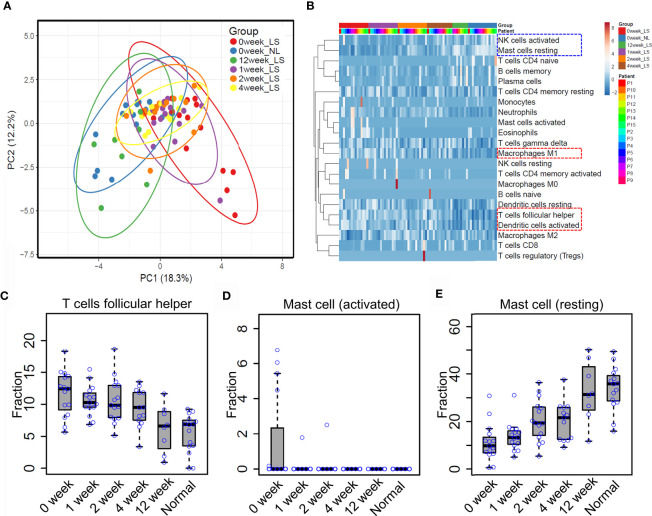
Mast cell homeostasis disruption may contribute to skin inflammation. **(A)** Principle component analysis (PCA) of the immune cell infiltration of non-lesional and lesional skin with etanercept treatment for 0, 1, 2, 4 and 12 weeks. **(B)** hierarchical clustering analysis of immune cell infiltration during the treatment. The fraction of the T follicular helper cells **(C)** activated mast cells **(D)** and resting mast cells **(E)** in non-lesional skin and lesional skin upon etanercept treatment for 0, 1, 2, 4 and 12 weeks.

Previous reports showed that etanercept treatment significantly improved the severity of psoriatic skin through targeting the TNF alpha pathway (19, 31). However, the dynamic of immune cell infiltration during the treatment remains almost unknown. To that end, we explored the proportion of different immune cell types in the lesional skin from psoriasis patients upon etanercept treatment (see *Methods and Materials*, **Supplementary Table S1**). As a result, PCA analysis showed that the immune cell infiltration of lesional skin with etanercept treatment for 12 weeks is similar with non-lesional skin, indicating that the infiltration of the immune cells represents the efficacy of etanercept treatment (**Figure 4A**). Further hierarchical clustering analysis demonstrated that the resting mast cells and the activated nature killer cells are enriched in non-lesional skin and lesional skin upon etanercept treatment for 12 weeks (**Figure 4B**, blue rectangle), while the activated dendritic cells, T follicular helper cells and macrophage M1 are enriched in lesional skin before or in early treatment for 1 week, 2 weeks and 4 weeks (**Figure 4B**, red rectangle and **Supplementary Table S5**). Particularly, the proportion of T follicular helper cells gradually decreases upon etanercept treatment for 12 weeks. The lesional skin has a similar fraction of T follicular helper cells with the non-lesional skin at the end of etanercept treatment (**Figure 4C**). Interestingly, we found that the activated mast cells were observed in lesional skin and quickly depleted upon etanercept treatment, indicating that the activated mast cell promotes the inflammatory responses in psoriatic skin (**Figure 4D**). Meanwhile, the proportion of the resting mast cells is gradually increased upon etanercept treatment, suggesting that the resting mast cell may play an important role in skin homeostasis maintenance (**Figure 4E**). In conclusion, our comprehensive analyses demonstrated the first immune cell landscape dynamics upon the treatment of TNF alpha inhibitors and highlighted the activated mast cells can promote inflammatory responses, while the resting mast cells may maintain skin homeostasis.

### Experimental Validation of Mast Cell Activation in Psoriatic Skin

**Figure 5:**
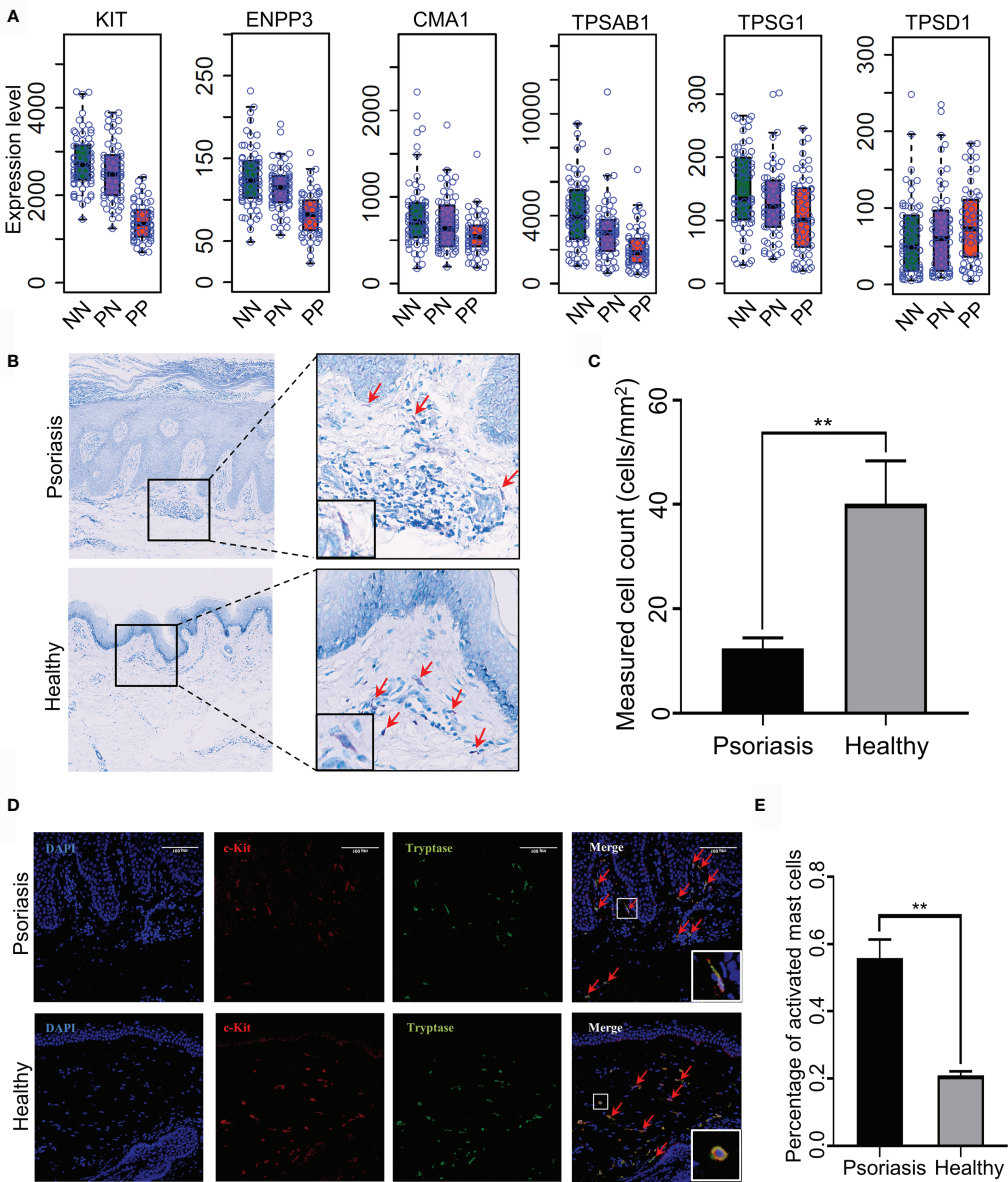
Experimental validation of mast cell activation in psoriatic skin. **(A)** Universal marker genes expression for mast cells in skin tissues. **(B, C)**. Toluidine blue staining shows that the population of mast cells is decreased in psoriasis skin compared with healthy controls. Red arrowheads indicate mast cells with toluidine blue staining. **(D)** Immunofluorescence (IF) staining demonstrates activated mast cell is increased in psoriasis skin compared with healthy controls. Red represents c-Kit and green represents tryptase. Red arrowheads indicate activated mast cells with double staining and a spindle-shaped morphology. The white rectangles indicate the representative activated mast cell (the top panel) and non-activated mast cell (the bottom panel). **(E)**. The proportion of activated mast cells in psoriatic skins compared with the healthy skin. N≥6 per group. **P < 0.01.

Recent reports demonstrated that T-helper cell type 17 (Th17) cells, natural killer (NK) T cells and plasmacytoid DCs might contribute to the hyper-inflammatory responses in psoriasis (31, 32). However, whether mast cells play an essential role in the pathogenesis of psoriasis remains mostly unknown. To this end, we collected skin biopsies and analyzed the universal marker genes expression for mast cells in psoriatic lesional and healthy skin. As a result, we found that the mRNA expression level of KIT, ENPP3, CMA1, CTSG, TPSAB1 and TPSG1 is decreased in PP compared with PN and NN, indicating the fraction of total mast cells is decreased in psoriasis (**Figure 5A**). However, we cannot determine the changes of the activated and resting state because these marker genes are consecutive expressed in mast cells (33). Particularly, we observed that the MCTC (Mast Cells with both Tryptases and Chymases positive) marker genes CMA1 is significantly down-regulated in PP (p-value < 0.05), again suggesting the dynamics of mast cell states in psoriatic skin may contribute to the pathogenesis of psoriasis (**Figure 5A**) (34). To further validate the participation of mast cells in psoriasis incorporating the staining and the cell morphology, we first confirmed that the population of mast cells is decreased in psoriasis compared with healthy controls using toluidine blue staining; the cells stained in purple are defined as mast cells (**Figures 5B, C**). As no experimental validated specific marker gene may be used to distinguish activated and resting mast cells, we incorporated c-Kit and tryptase immunofluorescence (IF) double staining and the morphology information to detect activated mast cells. Then, the cells with double-positive staining and a spindle-shaped morphology were defined as the activated mast cells (**Figure 5D**, indicated with red arrowheads). Expectedly, a significantly higher proportion of activated mast cells is observed in psoriatic skin compared with healthy skin (**Figures 5D, E**). Our integrative analyses demonstrated that mast cells are activated in psoriatic skin and may play a pro-inflammatory role in psoriasis through altering the immune cell niche in the skin.

## Discussions

Psoriasis is a chronic inflammatory skin disorder strongly associated with immune cell infiltration (31, 32). Thus, profiling the immune cell infiltration benefits our understanding of the pathogenesis of psoriasis. In the present study, we take advantage of high throughput transcriptomic datasets to infer the immune cell infiltration in the lesional and non-lesional skin tissue derived from psoriasis patients and healthy donors. Expectedly, we found that the immune cell profile of psoriasis and healthy skin is dramatically different in lesional compared with non-lesional skins, including activated mast cells, activated dendritic cells, activated CD4+ memory T cells, macrophage M0, macrophage M1 and neutrophils. Particularly, mast cell is activated in lesioned skin compared to non-lesioned skin. In contrast, mast cell activation is reduced upon etanercept treatment. Further, we validated that mast cells are indeed activated in psoriatic skin using immunofluorescence staining, which provides new insights into the pathogenesis mechanism of psoriasis.

The significance of this study is multi-folds. Firstly, we depicted a whole picture of immune cell infiltration of psoriatic skin through systematically integrative analysis of expression profiles from a large cohort of samples. Our study demonstrated that the alternation landscape of immune cell infiltration in psoriasis (**Figure 2B**). For instance, the activation of multiple immune cell types plays an important role in the pathogenesis of psoriasis, consistent with previous studies (31, 32). Secondly, our analysis demonstrated that deconvolution of immune cell proportions from expression profiles is reliable for investigating immune cell dynamics upon clinical treatment. Even though single-cell RNA-seq technologies have been widely used in clinical research, the high cost of scRNA-seq restricts its applications in large-scale clinical applications. Thus, our study provides a prototype for investigating immune cell infiltration in a large cohort of clinical samples. In addition, our analysis demonstrated the dynamic profiles of the resting and activated mast cells in lesional skin derived from psoriasis patients upon etanercept treatment, indicating that the computational deconvolution method has been achieved a high resolution for investigating cell composition (**Figures 4D, E**). Further analysis showed that mast cells could be identified through scRNA-seq using the drop-seq protocol. However, the proportion is low and tends to capture in-mature mast cells, thus failing in studying the resting and activated mast cells in lesional skin of psoriasis (**Supplementary Figure S1**). We believe that these advantages of computational deconvolution of immune cell infiltration would accelerate the mechanistic studies in other skin diseases.

The mast cell is a long-lived cell located at the upper dermal skin, which is activated and recruits other immune cells to induce an inflammatory response in psoriasis (32, 35). The current study observed that mast cell is activated in lesioned skin but with a resting state in healthy skin. Therefore, we suspected that mast cells might activate and degranulate during the progression of psoriasis and finally lead to chronic inflammation. To validate our hypothesis, we profiled the immune cell infiltration dynamics in skin tissue upon etanercept treatment. Interestingly, mast cells gradually return to the resting state during the treatment, indicating the important role in the progression of chronic inflammation and skin cell niche maintenance. In summary, the immune cell infiltration obtained in the current study is useful for further immunological studies in skin disease. We highlight the important functions of mast cells during the progress of psoriasis.

## Data Availability Statement

The original contributions presented in the study are included in the article/**Supplementary Material**. Further inquiries can be directed to the corresponding authors.

## Ethics Statement

The studies involving human participants were reviewed and approved by Department of Dermatology, Dermatology Hospital of Southern Medical University. The patients/participants provided their written informed consent to participate in this study.

## Author Contributions

Conceived and designed the experiments: JZ and BY. Performed the experiments: YS and YZ. Analyzed the data: JL and XL. Wrote the paper: JZ and YS. Reviewed and edited the manuscript: JZ and BY. All authors contributed to the article and approved the submitted version.

## Funding

This study is substantially supported by National Natural Science Foundation of China (NSFC): 31900447 and 32070792; Startup foundation of dermatology hospital, Southern Medical University (2019RC06) and Guangzhou Municipal Science and Technology Bureau (202102020719).

## Conflict of Interest

The authors declare that the research was conducted in the absence of any commercial or financial relationships that could be construed as a potential conflict of interest.

## Publisher’s Note

All claims expressed in this article are solely those of the authors and do not necessarily represent those of their affiliated organizations, or those of the publisher, the editors and the reviewers. Any product that may be evaluated in this article, or claim that may be made by its manufacturer, is not guaranteed or endorsed by the publisher.
