# Supplementary figures and images for: Immune Cell Infiltration Analysis Demonstrates Excessive Mast Cell Activation in Psoriasis
Source: Front Immunol. 2021 Nov 23;12:773280. doi: 10.3389/fimmu.2021.773280 (PMC8650163; doi:10.3389/fimmu.2021.773280)

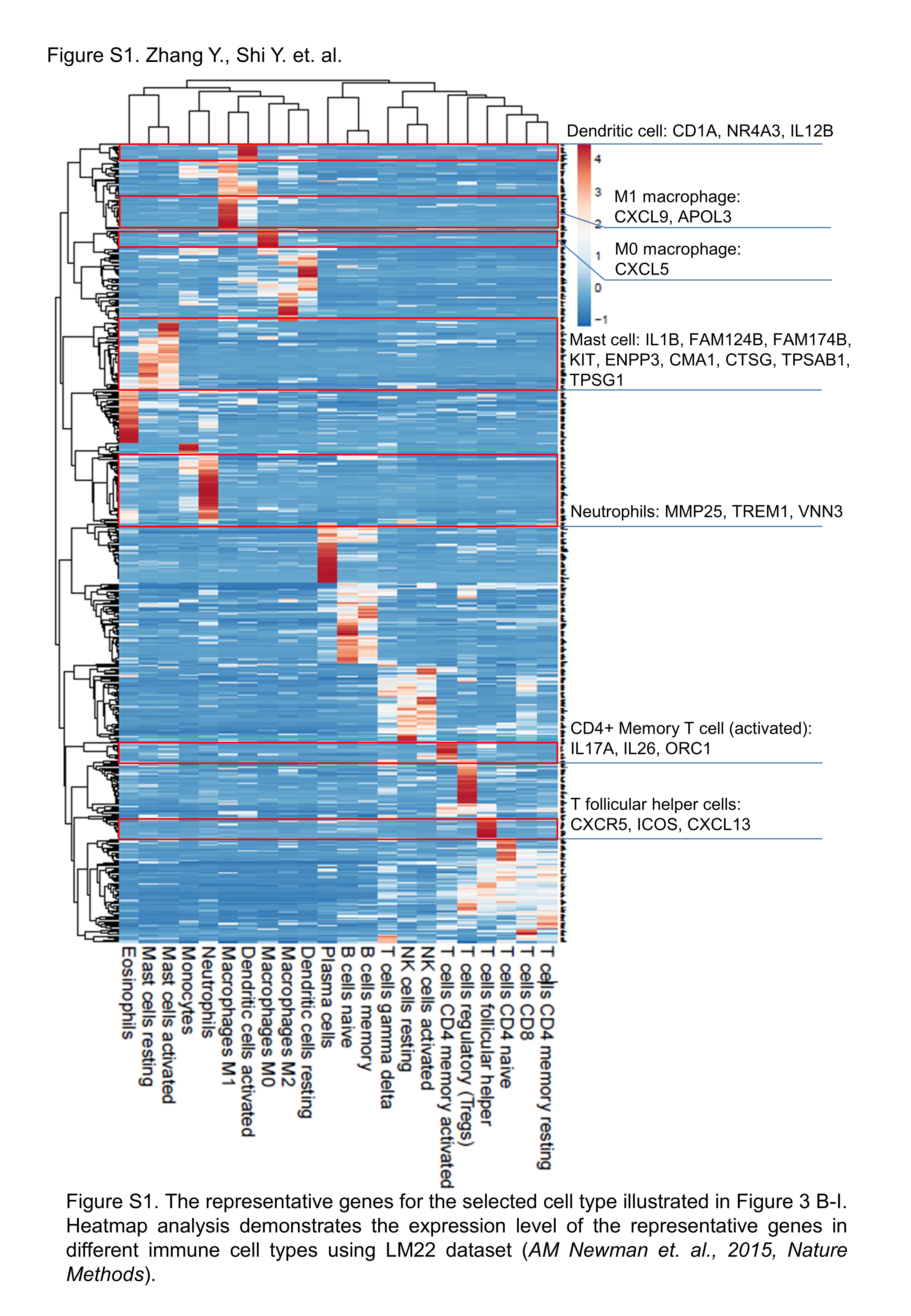

Supplement: Supplementary file 1 [file Image_1.tif]

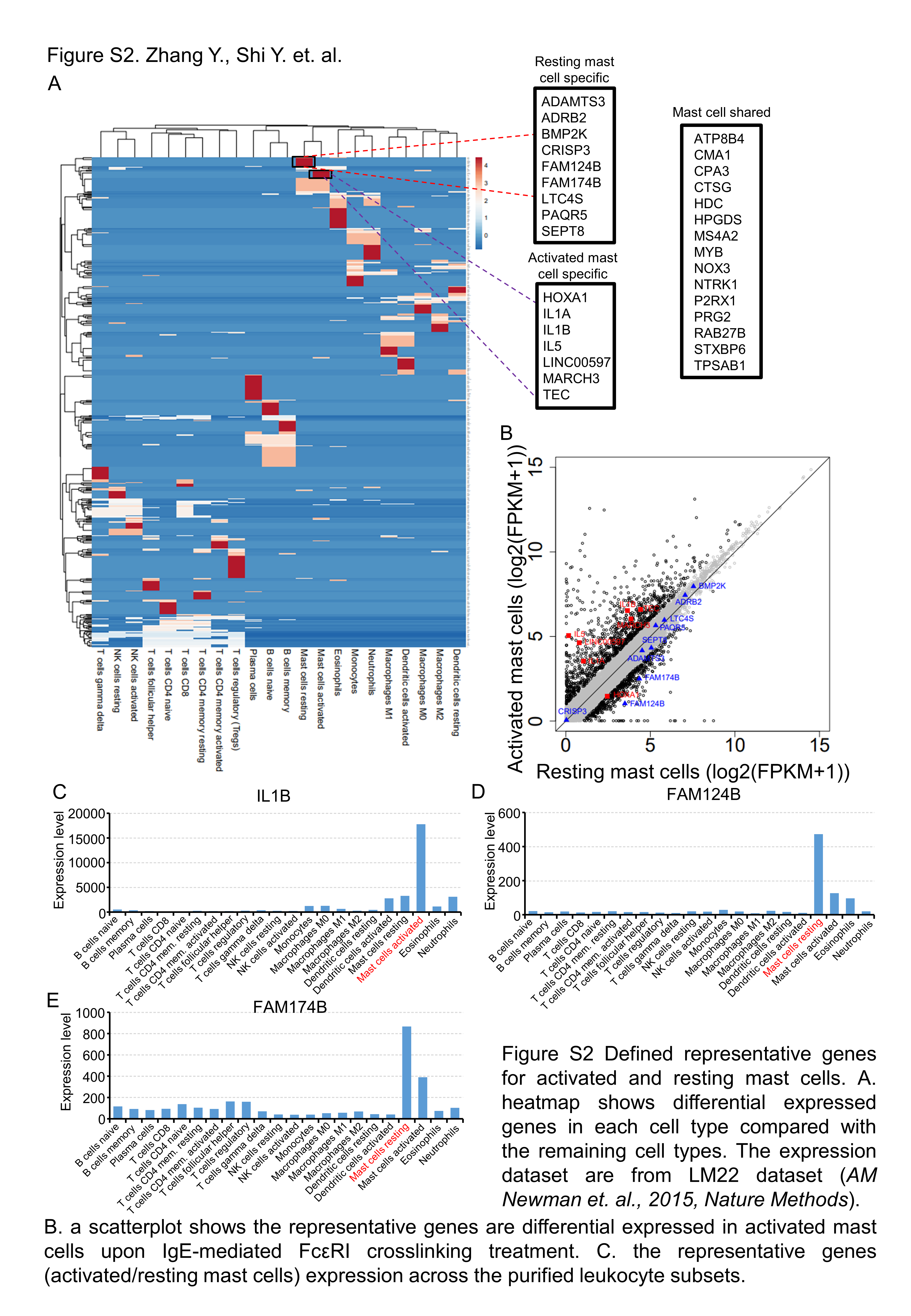

Supplement: Supplementary file 2 [file Image_2.tif]

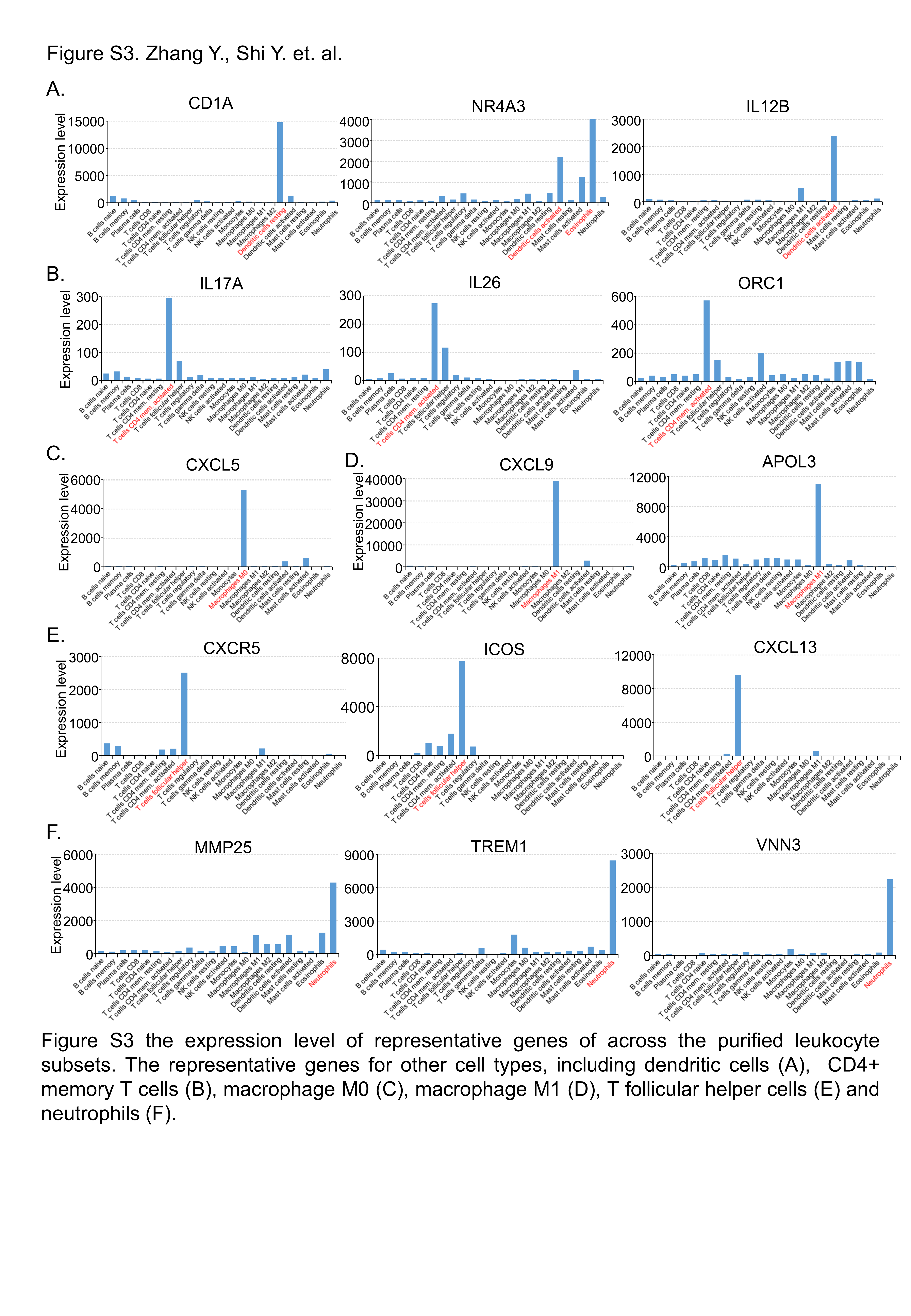

Supplement: Supplementary file 3 [file Image_3.tif]
